# *Lychaete pellucida* as a novel biosorbent for the biodegradation of hazardous azo dyes

**DOI:** 10.1007/s10661-023-11518-w

**Published:** 2023-07-11

**Authors:** Hussein A. Khalaf, Mostafa M. El-Sheekh, Mofida E. M. Makhlof

**Affiliations:** 1 Chemistry Department, Faculty of Science, Damanhour University, Damanhour, Egypt; 2 Botany Department, Faculty of Science, Tanta University, Tanta, 31527 Egypt; 3 Botany and Microbiology Department, Faculty of Science, Damanhour University, Damanhour, Egypt

**Keywords:** *Lychaete pellucida*, Reactive azo dyes, Biosorption, Dye removal, SEM, FTIR, Isothermal, Kinetic study

## Abstract

The majority of textile wastes are made up of toxic dyes. Additionally, because these compounds are soluble, wastewater may include significant concentrations. In this work, the green alga *Lychaete pellucida* is used for the bioremoval of four common azo dyes, Reactive Blue 4 (RB4), Reactive Red 120 (RR120), Reactive Brilliant Yellow 3G (RBY3G), and Reactive Green12 (RG12), with the application of two models of sorption isotherms, Langmuir and Freundlich. The spectrophotometer method was used to identify optimum conditions (temperature, pH, dye concentrations, algal biomass, and contact time) to remove these dyes onto dry freshwater macroalgae. The optimum pH for *L. pellucida* was 8. The optimum biosorbent amount is 2 g/L. Then, the best-removed dye concentration was 5 mg/L, the optimum contact duration was 120 min, and the optimum temperature was 25 °C. Under optimum conditions, the percent of dye removal was about 95% for all used azo dyes. This is the first report on the use of *Lychaete pellucida* for the efficient biodegradation of hazardous azo dyes*.*

## Introduction

Nowadays, nearly all businesses, from the food to the pharmaceutical industry, employ a wide variety of synthetic colors extensively (Aleem, 1993; Keskinkan et al., 2004; Slama et al., 2021). Colorants can be natural or synthetic and are utilized in many industries, including pulp, paints, tanneries, and garments. Since many years ago, significant amounts of synthetic dyes have been emitted globally, posing risks to the environment and human health. According to reports (Aksu & Tezer, 2005; Sarojini et al., 2022), 10 to 15% of the dye used in the production of textile items is discharged into the environment each year. These colors harm vegetation, animals, and human life when they are released into headwaters. Certain colorants can induce allergies, skin conditions, cancer, and mutations in humans and animals. Additionally, these dyes bring on the absorption and reflection of sunlight. The fundamental issue is that dyes promote the growth of bacteria, which makes it challenging for them to break down in water (Al-Tohamy et al., 2022; Wu & Wang, 2001).

They are water-soluble synthetic colorants with a polyaromatic formation; however, 40% of these colors end up as waste due to their ability to react with the hydroxyle group (El-Sheekh et al., 2021; Mallakpour et al., 2023). When coloring wool and silk, reactive colorants are most frequently used. Binding the reactive sides of molecules is the primary method. The method of binding is through covalent bonds. Chromophore moiety is a common property of reactive dyes. Both aerobically and anaerobically, these kinds of pigments degrade slowly and produce carcinogenic aromatic amines as byproducts (Abdoul et al., 2023; Safi et al., 2014).

Azo, anthraquinone, and phthalocyanine dyes are the three most popular categories of reactive dyes (Dotto et al., 2012; El-Sheekh et al., 2022). Only one diazonium molecule is commonly combined with phenol or an aromatic amine to create the azo-dye organic compounds (Junnarkar & Pandhi, 2017). The greatest and most diverse classes of colors are employed in various sectors, including laser coloring, textile, sheet, and fiber. Typically, 31–69% of the azo colors used in the coloring process are hydrolyzable and mixed with wastewater (Dotto et al., 2012). Additionally, inappropriate waste dye disposal in water poses a serious risk to human health (Junnarkar & Pandhi, 2017). Therefore, it is crucial for the ecology that azo dyes are successfully removed from wastewater (Dotto et al., 2013). Various methods can be used to remove azo dyes from wastewater (Al-Tohamy et al., 2022). The vast majority of the time, extremely costly natural, physical, and chemical techniques are utilized to clean up excess material. To remove contaminants, processes including photocatalytic analysis, chemical oxidation, and anaerobic/aerobic biological treatment are used (Acuner & Dilek, 2004; Verma & Kumar, 2023). However, most technologies cannot be used since they are costly and produce additional pollution (Khataee et al., 2013). The organic compounds that make up the dyes used in the textile industry have a variety of functional groups, making conventional techniques of treating them challenging. Compared to other methods of dye removal, the adsorption process is the most efficient method for removing contaminants from wastewater since it has numerous advantages for the environment and the economy (Ali et al., 2020). In this context, biosorption is an inexpensive and efficient way to remove paint from industrial surfaces. It is a physicochemical process in which contaminants are removed from wastewater using biosorbents, including fungi, bacteria, and algae (El-Sheekh et al., 2009; Mitrogiannis et al., 2015).

Red algae, green algae, and brown algae are three categories of multicellular algae, including macroalgae. As non-living biomasses, macroalgae can be utilized to remove different textile colors. Consequently, macroalgae are non-toxic, inexpensive, and widely accessible biomass for colored effluent treatment with varying success degrees (Aziam et al., 2021; El-Sheekh et al., 2022). Biosorption of colorants can be performed using both living and non-viable algae with the availability of recovery and reusing biomass (Singh & Kaur, 2013). The algae’s surface can absorb impurities from effulents (El-Sheekh et al., 2022). Marungrueng and Pavasant (2007) looked into the dye’s ability to adhere to the green macroalga *Caulerpa lentillifera*. When their findings were contrasted with the commercial activated carbon’s ability at sorption, it became clear that the algae had higher dye sorption capabilities than the activated carbon. Because proteins, polysaccharides, and lipids serve as binding sites on the surface of macroalgae cell walls, they have a high binding affinity (Abd Ellatif et al., 2021; Davis et al., 2003). The pollutant’s chemical characteristics, the material used, and the environment all impact biosorption (Vijayaraghavan & Yun, 2008). A macroalga belonging to the chlorophyte class is *L. pellucida*, a fresh and marine green alga. This green alga’s main identifying features include a true branched filament that forms a net-like structure (Fayyad et al., 2022). Then, incredibly stiff filament often remains stable in a single location, making it simple to collect (Lefta et al., 2022). As a result, and for the first time, our study focuses on how to remove toxic, carcinogenic reactive dyes (Reactive Blue 4 (RB4), Reactive Red 120 (RR120), Reactive Brilliant Yellow 3G (RBY3G), and Reactive Green12 (RG12) from aqueous solution by using a local isolate of the green alga, *Lychaete pellucida* (Hudson) (Wynne, 2017), as a free biosorbent. The effects of unique operational parameters such as pH, temperature, contact time, algal weight, and beginning dye concentration have been investigated in batch biosorption experiments. The non-living *L. pellucida* adsorption isotherms and kinetics have also been investigated.

## Material and methods

### Algal sampling and preparation

In the winter of 2023, samples were collected from the drainage basin of agricultural land in the hamlet of Edfina in the El Beheira Governorate of Egypt, located at latitude 31° 17′ 36″ N and longitude 30° 30′ 45″ E. The specimens were pressed and stored in a 4% formalin solution to help identify the green macroalga, then identified according to Aleem (1993), and second, double-check the identification and habitat information by the Algae Base website. Samples of the green macroalga were manually collected separately from the basin, packed within sealed plastic bags, and transported immediately to the laboratory (Guiry & Guiry, 2020). The alga was thoroughly cleansed three times with new distilled water to remove sticking materials like clay, grass, shells, and other things. The macroalga was dried for 40 days in a darkened lab area before being heated to 50 °C. The algal parts were ground to a fine powder using an electronic blender after they had dried completely. Then they were weighed and stored in sterile plastic sheets.

## Dye preparation

Four reactive dyes are used for this study; their names and characters are cited in Table 1. When necessary, the stocks of the tested colors were prepared with a concentration 1000 mg L^−1^.

**Table 1:** Chemical properties of the used reactive azo dyes

Dye name	Reactive Red 120	Reactive Blue 4	Cibacron Brilliant Yellow 3G (Reactive yellow)	Reactive Green 19
Abbreviation	RR120	RB198	RBY3G	RG19
Dye type	Reactive	Reactive	Reactive	Reactive
λ max	500	600	410	660
M.wt	1338.09	637.43	872.96	1418.93
CAS number	61951–82-4	13324–20-4	50662–99-2	61931–49-5
Color index	292775	61205	18972	205075
Molecular formula	C44H30Cl2N14O20S6	C23H14Cl2N6O8S2	C25H15Cl3N93O10S3	C40H23Cl2N15Na6O19S6

## Algal characterization

### FTIR

FTIR analyses were carried out on KBr discs and analyzed in an FTIR spectrophotometer, FTIR-6100 type A, JASCO International Co., Ltd. Spectra were acquired between 4000 and 400 cm^−1^, and spectral data were analyzed using Spectrum software at a resolution of 4 cm^−1^, to identify the surface functional groups of the *L. pellucida* (LP) biomass that are involved in dye adsorption.

## SEM

*L. pellucida* surface morphology was monitored before and after dye removal by a scanning electron microscope (JEOL-JSM-5200 LV, USA).

## Biosorption study

### Batch biosorption study

The biosorption process was applied to remove four reactive dyes which are highly used in the textile industry. These dyes are Reactive Blue 4 (RB4), Reactive Red 120 (RR120), Cibacron Brilliant Yellow 3G (RY3G), and Reactive Green19 (RG19), and their chemical structures and other information are cited in Table 1. Dye stock solutions were prepared in concentration 40 mg L^−1^, and λmax dyes were measured by UV–Vis Spectrophotometer (Thermo Scientific™ Evolution 300), between 200 and 700 nm. To study the effect of different factors in dye removal, a range of pH (2, 4, 6, 8, 10), algal biomass weight (0.5–3.0) mg L^−1^, dye concentration (1–7) mg L^−1^, temperature (25–55 ℃), and contact time (20–180) min, was investigated (Waqas et al., 2015). The capacity of dye removal in water (*R*%) is calculated by Eq. 1:

1 $$R\,{ \% }= \frac{{C}_{\mathrm{o}} -{C}_{\mathrm{e}}}{{C}_{\mathrm{o}}} \times 100$$

where *C*_o_ and *C*_e_ are the initial and equilibrium dye concentrations, respectively.

## Biosorption isotherms and kinetic studies

To ascertain the correlation between biosorption capacity and dye concentration at equilibrium, two sorption isotherm models, Langmuir (1918) and Freundlich (1907), were presented. Both Langmuir and Freundlich models are represented by Eqs. 2 and 3.

2 $$\frac{{C}_{\mathrm{e}}}{{q}_{\mathrm{e}}}=\frac{1}{{K}_{\mathrm{L}}}+\frac{{a}_{\mathrm{L}}}{{K}_{\mathrm{L}}}{C}_{\mathrm{e}}$$

3 $$\log{q}_{\mathrm{e}}=\log{K}_{\mathrm{F}}+\frac{1}{n}\log{C}_{\mathrm{e}}$$

where *C*_e_ is the equilibrium dye concentration (mg L^−1^), *q*_e_ is the adsorption capacity (mg g^−1^), and *a*_L_ and *K*_L_ are the constants of Langmuir, while *K*_F_ is the Freundlich constant and *n* is the Freundlich exponent.

For the purpose of explaining how dyes are transported onto various adsorbents, kinetic equations have been constructed. To identify the biosorption mechanism, such as mass transfer and chemical reaction, two kinetic models—pseudo-first order and pseudo-second order—were used in this investigation (Khalaf, 2014; Nandi et al., 2009). Equations (4) and (5) represent the pseudo-first and pseudo-second-order models, respectively.

4 $$\mathrm{log}\left({q}_{\mathrm{e}}-{q}_{t}\right)=\mathrm{log}\,{q}_{\mathrm{e}}- \frac{{K}_{1}t}{2.303}$$

5 $$\frac{t}{{q}_{t}}= \frac{1}{{K}_{2 }{q}_{\mathrm{e}}^{2}} + \frac{t}{{q}_{\mathrm{e}}}$$

where *q*_e_ represents the amounts of adsorbed dye (mg g^−1^) at equilibrium,*q*_*t*_ is the amount of adsorbed dye (mg g^−1^) at time *t* (min), *k*_1_ is the pseudo-first-order rate constant (min^−1^), and*k*_2_ is the pseudo-second-order rate constant (g mg^−1^ min^−1^).

## Results and discussion

### Algal FTIR spectrum

**Fig. 1:**
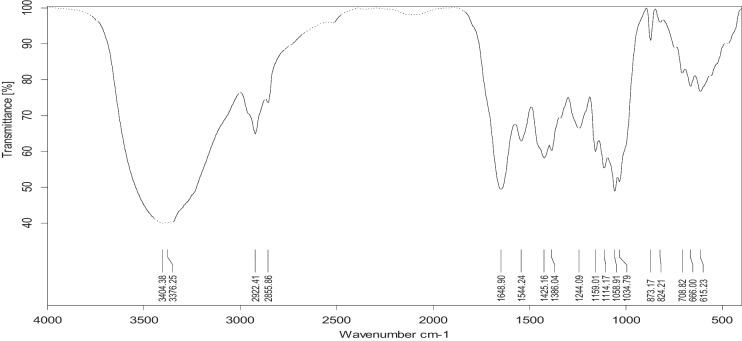
FTIR spectrum of *L. pellucida* algae before adsorption

**Fig. 2:**
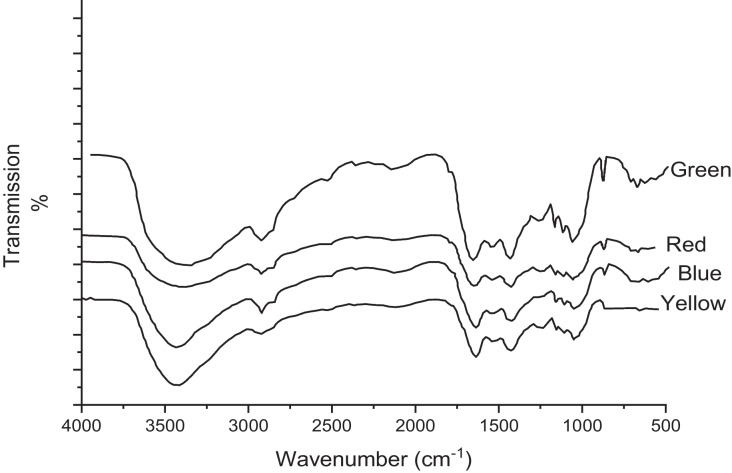
FTIR spectrum of *L. pellucida* algae after green, red, blue, and yellow reactive dye adsorption

Figures 1 and 2 show the FTIR spectrum of *L. pellucida* algae in the range 4000 to 400 cm^−1^ before and after biosorption. The band at wavenumbers of 3700 to 3000 cm^–1^ for algae represents OH groups on the biosorbent surface. The band that occurred at 2922 cm^–1^ and 873 cm^–1^ represents C–H groups. The 1650-cm^–1^ band is responsible for the CO stretching mode, conjugated to an NH deformation mode, and is indicative of an amide band. The band at 1159 cm^–1^ is due to CO or CN groups. Small peaks at 1114 cm^−1^ may belong to secondary and tertiary alcohol CO, and a peak at 1425 cm^−1^, which the methylene group may result. These FTIR data reveal the presence of several functional groups that can be bound with reactive dyes on the *L. pellucida* algal surface, as we found shifting in some breaks in FTIR analysis after adsorption referring to the attachment of the dyes with the algal surface-active groups. Furthermore, one can notice that the *L. pellucida* spectrum after dye biosorption displayed an increment in peak intensity (not shown) than that before dye adsorption. According to Omar et al. (2018), the interactions between algal biomass and dye can be explained as follows: (1) the interaction of the cationic dye with the carboxylate group, (2) formation of coordination between dye and the lone pair of electrons of the OH or NH_2_ group, (3) Van der Waals forces between the nonpolar groups of the dye and the algal cell, and (4) the ion–dipole bond formed by the dye molecule and the negative dipole end of the CO_3_^−2^ group.

## SEM images

**Fig. 3:**
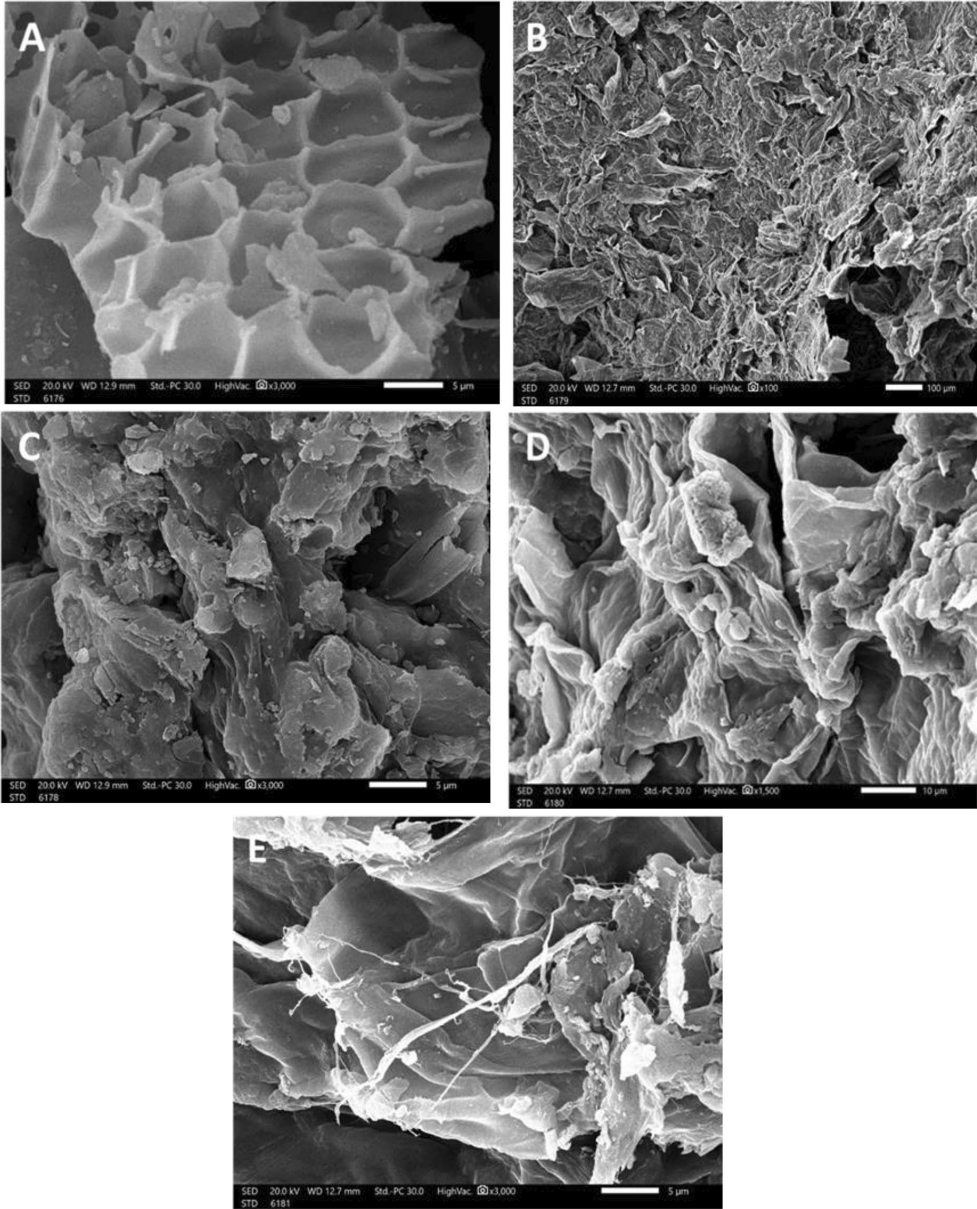
Scanning electron microscopy of the surface of *L. pellucida* before (**A**) and after green (**B**) yellow (**C**) red (**D**), and blue (**E**) azo dye removal

The applied SEM analysis explained the changes in *L. pellucida* surface before and after dye adsorption, as shown in Fig. 3A–E. Results showed that before being submerged in various colors, algal cells had a smooth, extremely porous structure that was hole-like. The surface of the *L. pellucida* biomass changed after being exposed to the dye due to the precipitation of dye molecules around the biomass surface, making it rough and meandering (Fig. 3B–E).

## Batch biosorption equilibria

### Contact time and initial concentrations

**Fig. 4:**
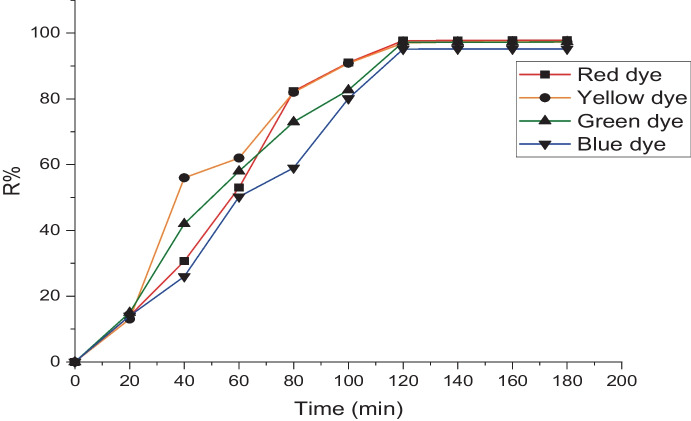
Removal % of different dyes under the effect of contact time by using *L. pellucida* algae biomass

**Fig. 5:**
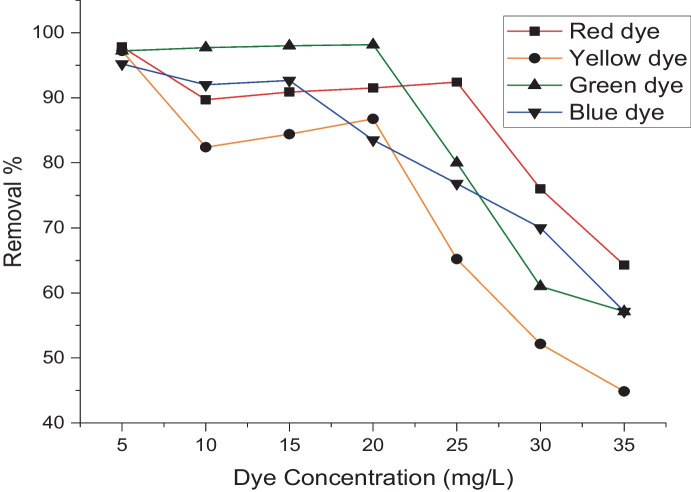
Removal % of different concentrations of dyes by using *L. pellucida* algae biomass

Performance and cost are constantly in charge of adsorption process efficiency. As a result, the two most crucial characteristics that can be comprehended in an adsorption system are the adsorption capacity and contact time. These tests were conducted with 0.005 g of biomass and an initial dye concentration of 5 mg L^−1^ at 25 °C for an equilibrium time of 180 min. The adsorption process was rapid at the first hour but reached equilibrium after 100 min. Figure 4 shows the amount of adsorbed dye, suggesting that with increasing time, dye removal increases. After 120 min, removal for the red, yellow, green, and blue adsorbed dyes stabilizes at 95, 96, 97, and 97%, respectively. All equilibrium trials, however, were permitted to run for 3 h. The amount of dye adsorbed remained nearly constant up to 20 mg L^−1^ 90, 88, 97, and 82% for red, yellow, green, and blue adsorbed dyes, respectively, before decreasing with increasing the dye concentration (Fig. 5). Additionally, the concentration of the adsorbed dye increased (5 up to 35 mg L^−1^). The effectiveness of the process of adsorption and the effectiveness and applicability of a mathematical model are determined by the solid phase’s adsorption capacity.

## Effect of pH

**Fig. 6:**
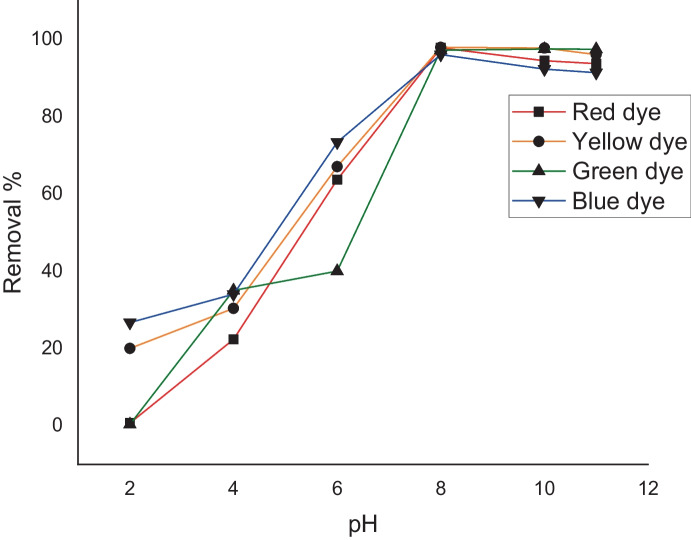
Removal % of different dyes under the effect of pH values by using *L. pellucida* algae biomass

The 5-mg L^−1^ initial dye concentration and 0.005 g of algal mass at 25 °C for 180 min were used. The effect of initial pH (2, 4, 6, 8, and 10) was tested on the biosorption from multi-aqueous solution onto algal surfaces (Fig. 6). The maximum percent of removal was 97% for red, yellow, and green dyes and 96% for blue dye, obtained at basic pH (pH = 8) with little decrease in the removal of dyes after pH 8. The improvement in reactive dye adsorption at basic pH was due to the electrostatic interaction between the anionic groups on the reactive dyes and the + ve surface of the alga. The negative electrical structure of the chromophore group is the reason why reactive dyes are also known as anionic dyes (Khalaf, 2008). The amount of -ve sites on the *L. pellucida* surface rises as pH rises, whereas the amount of positively charged sites falls. Due to electrostatic repulsion, a negative surface charge (in basic media) inhibits the adsorption of reactive dyes (Anbia & Ghaffari, 2011; Namasivayam & Kavitha, 2002).

## Temperature effect

**Fig. 7:**
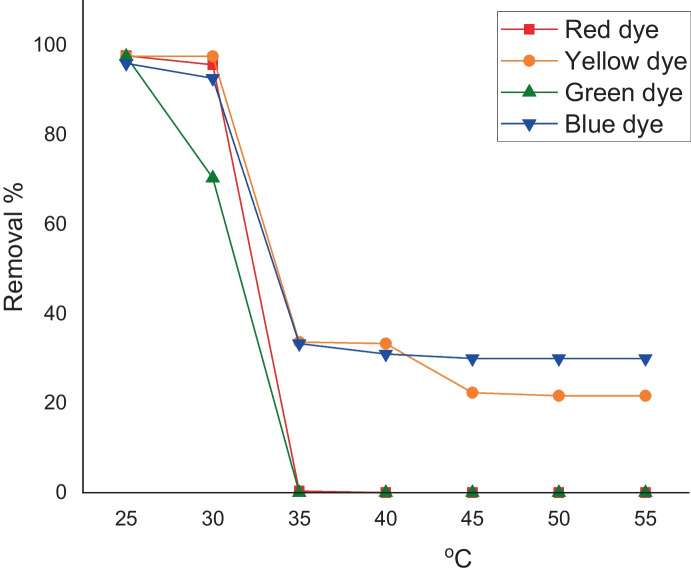
Removal % of different dyes under the effect of temperatures by using *L. pellucida* algae biomass

Temperature effect was studied at the same mentioned conditions, pH 8, and at different temperatures (from 25 to 55 °C) with shaking for 180 min. Figure 7 illustrates that the biosorption rate decreased as the temperature increased. The maximum dye removal (more than 96% for all reactive dyes) was obtained at 25 °C. An increase in the temperature causes a sharp decrease (at 35 °C) in the dye removal % to reach zero % for red and green dyes, 35% for blue, and 20% for yellow dye, which improves that this process is exothermic. Remazol Black B and Acid Red 274 dye biosorption have been observed to have an exothermic character (Mohan et al., 2002; Özer et al., 2005). The obtained data indicated that biosorption activity was thermally deactivated at temperatures greater than 30 °C. As a result, the *L. pellucida* biomass in use might adapt to temperatures up to 30 °C. According to reports, green algae could remove the most pigment at room temperature (Omar et al., 2018). This outcome might result from a weak interaction between molecules of dye and the active binding sites of the biomass at high temperatures, which leads to damage to the active binding sites of the biomass.

## Effect of *L. pellucida* loading

**Fig. 8:**
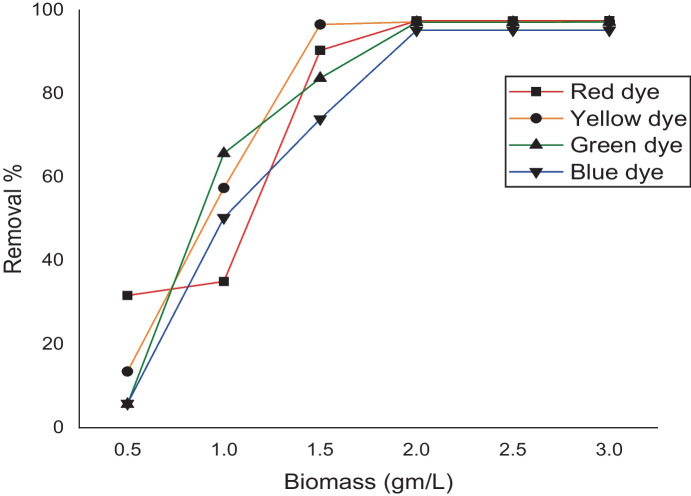
Removal of different dyes under the effect of weights of *L. pellucida* algae biomasses (g/L)

To study the effect of *L. pellucida* concentrations, the biosorption of the different dyes onto *L. pellucida* biomasses was measured at different biosorbent concentrations (0.5–3.0 g L^−1^). Our results showed that, as the *L. pellucida* concentration increased, dye removal % increased (Fig. 8) with maximum removal of 96.5% obtained for yellow dye at 1.5 g L^−1^ and about 97% for the red, green, and blue dyes obtained at a biomass concentration of 2 g L^−1^, and this in turn is due to the increment of the free adsorption site number that occurs with biosorbent concentration increment. Thus, the amount of dye removed is sharply increased with the biosorbent concentration until it reaches a certain biomass (2 g/mL) after which any increment in *L. pellucida* weight did not affect the dye removal rate.

## Biosorption isotherms

**Fig. 9:**
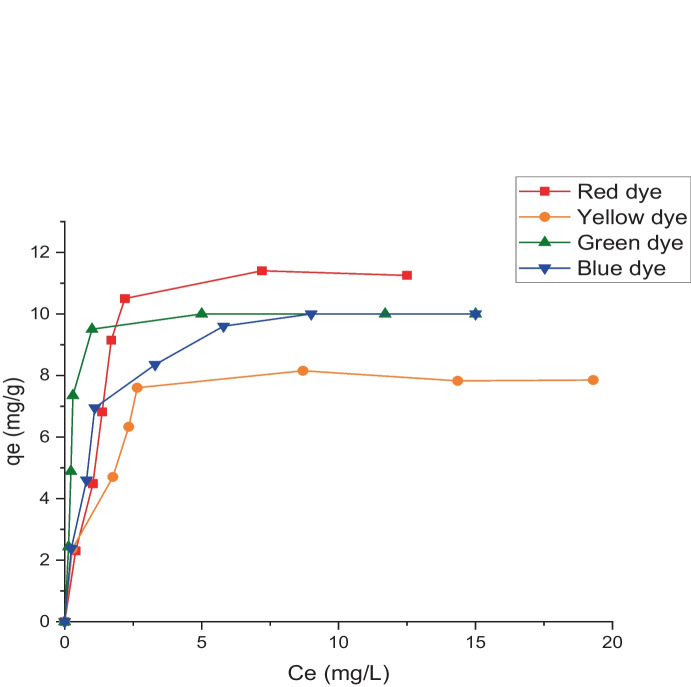
Biosorption isotherms of different dyes onto *L. pellucida* algae biomasses

**Fig. 10:**
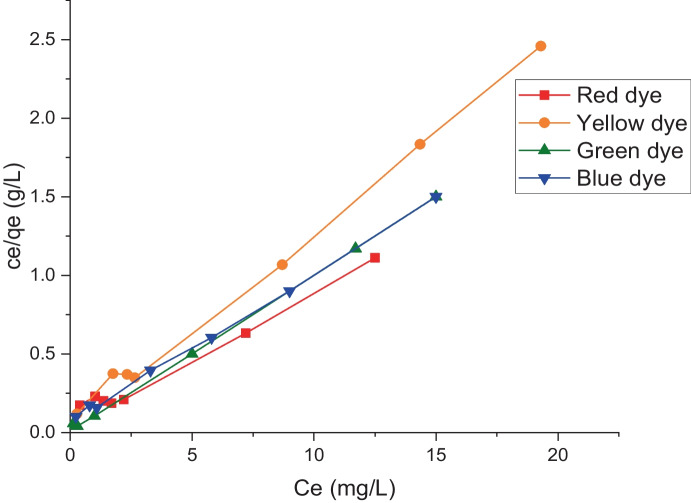
Langmuir adsorption isotherm for indicated dye biosorption onto *L. pellucida* algae biomass

Figure 9 shows the plotting of the experimental data points of the biosorption of tested dyes onto LP biomass, *q*_e_ (mg g^−1^) versus Ce (mg L^−1^). The resulting L shape of an isotherm represents both the potential mode of dye adsorption and information on the affinity of the dye molecules for adsorption. The type-L isotherm implies that these reactive dye molecules have a moderate affinity for the active sites of the biosorbent *L. pellucida* (El-Geundi, 1990). It is critical to analyze this isotherm in order to determine which models are suitable for use in design. In this investigation, two distinct isotherms were employed. Equation 2 can be used to express the first, Langmuir. For the adsorption of four reactive dyes onto the LP biosorbent, the linear plot of *C*_e_/*q*_e_ against *C*_e_ (Fig. 10) indicates the applicability of the Langmuir isotherm (*r*^2^ higher than 0.98) of the current systems and shows monolayer coverage of the adsorbate at the outer surface of the adsorbent (*c*). The results of the least-square approach were used to determine the values of *K*_L_ and *a*_L_, which are listed in Table 2. According to Table 2, the maximum capacities (*q*_e_) for the red, yellow, green, and blue dyes were 12.6, 8.2, 10.3, and 10.7 mg g^−1^. Through *R*_S_, the Langmuir constant (*a*_L_) was employed to demonstrate the affinity of the adsorbent to the adsorbate (dimensionless separation factor). *R*_S_ was expressed as follows (Elayappan et al., 2022; Khalaf, 2014):

**Table 2:** Parameters of Langmuir and Freundlich isotherms

Dye	Langmuir parameters							Freundlich parameters		
	*K*_L_ (L g^−1^)	*a*_L_ (L mg^−1^)	*q*_max_ (mg g^−−1^)	*q*_ref_ (mg g^−1^)	*C*_ref_ (mg L^−1^)	*R*_S_ ( −)	*r*^2^	*K*_F_ (L g^−1^)	*n*	*r*^2^
Red	10.7	0.85	12.6	11.2	12	0.09	0.98	5.09	2.3	0.73
Yellow	12.7	1.54	8.2	8.1	9.5	0.06	0.99	4.19	3.6	0.80
Green	50.3	4.9	10.3	10	12	0.01	0.99	6.6	4.6	0.63
Blue	13.5	1.26	10.7	10	10	0.07	0.99	4.9	2.9	0.87

6 $${R}_{\mathrm{S}}= \frac{1}{1+ {a}_{\mathrm{L}}{C}_{\mathrm{o}}}$$

The value of separation factor (*R*_S_) lies between 0 and 1 for favorable adsorption, while *R*_S_ > 1 represents unfavorable adsorption, and *R*_S_ = 1 represents linear adsorption while the adsorption process is irreversible if *R*_S_ = 0 (Khalaf, 2014).

The values of *R*_S_ for the tested reactive dyes onto the *L. pellucida* biosorbent were found between 0 and 1, indicating that the removal process of these dyes by using algal biomasses is favorable.

**Fig. 11:**
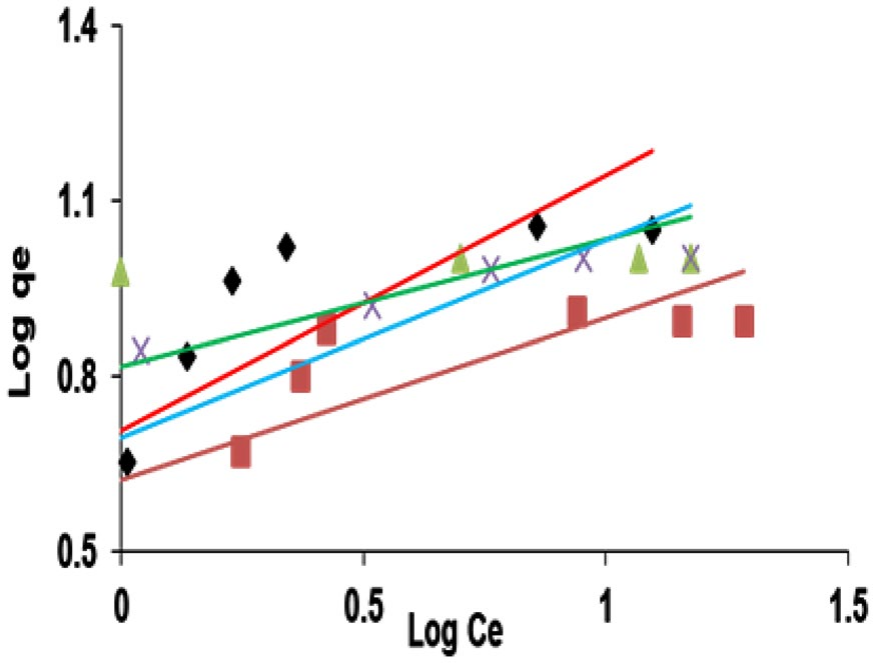
Freundlich adsorption isotherm for indicated dye biosorption onto *L. pellucida* algae biomass

A plot of log *q*_e_ against log *C*_e_ demonstrates some curvature, according to results from the second model, Freundlich isotherm, which may be seen by looking at the results (Fig. 11). The least-squares approach that applies to the straight lines in Fig. 10 was used to determine the Freundlich parameters, *K*_F_ and *n*, which are listed in Table 2 for the biosorption of the four dyes that were tested upon LP. This demonstrates that the *n* values are greater than one, showing that the examined dyes are effectively absorbed by the biomass of *L. pellucida*. Values where *n* > 1 represent favorable adsorption conditions. In most cases, the exponent between 1 < *n* < 10 shows beneficial adsorption (Ho & McKay, 2000). The correlation coefficients (*r*^2^) of the Langmuir and Freundlich isotherms are close to unity; however, the *r*^2^ of the Freundlich isotherm is significantly lower than that of the Langmuir isotherm (Table 2), which shows the findings of the obtained experiments. This demonstrates the applicability of both the surface and the Langmuir isotherm.

## Biosorption kinetics

**Fig. 12:**
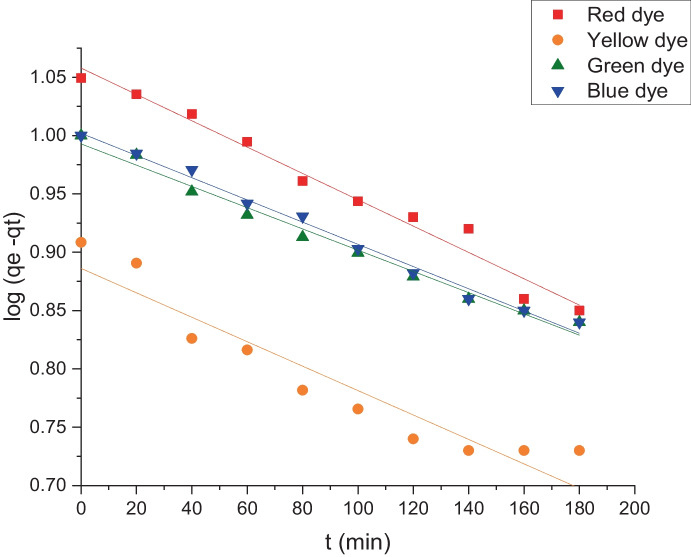
Pseudo 1st order for the indicated dye biosorption onto *L. pellucida* algae biomass

**Fig. 13:**
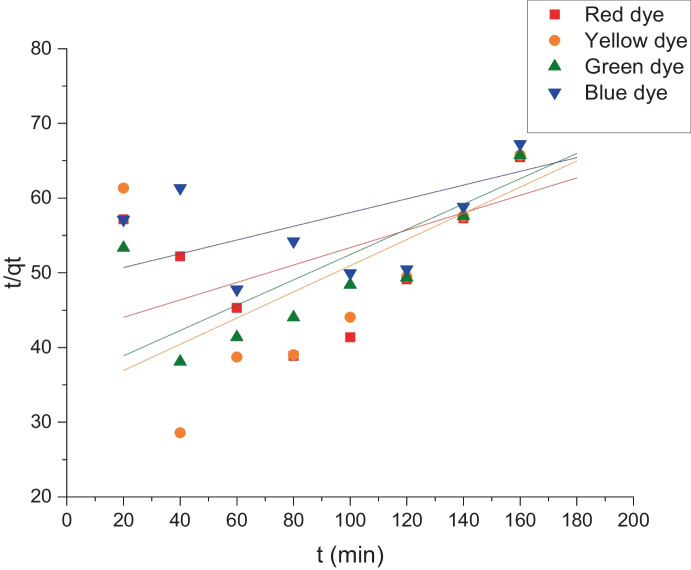
Pseudo 2nd order for the indicated dye biosorption onto *L. pellucida* algae biomass

Figures 12 and 13 expose the kinetic models, pseudo-first and pseudo-second-order, respectively. From these figures, it can be noticed that all dyes have high regression factors, *r*^2^, which were found to be more than 0.9 for the pseudo-1st-order kinetic model. Also, *q*_e_ calculated values were near the results of experimental values as shown in Fig. 12 and Table 3. On the other hand, the pseudo 2nd-order rate constant values (*k*^2^) were calculated from Eq. (4) as shown in Table 3 and Fig. 13. The regression factors (*r*^2^) values for the pseudo 2nd order found to be less than 0.9, and the (*q*_e_) calculated values did not fit with the experimental values which proved that the biosorption does not follow the pseudo 2nd order. Therefore, it can be said that the pseudo-1st-order model was followed throughout the biosorption of these four reactive dyes. When the rate of occupation of the adsorption sites is proportional to the number of unoccupied sites on the adsorbent, the pseudo-first-order kinetic model is employed (Miyah et al., 2017).

**Table 3:** The kinetic data according to pseudo-first and pseudo-second order

Dye	Pseudo 1st order			Pseudo 2nd order		
	*k*_1_ (min^−1)^	*q*_e_ (mg g^−1^)	*r*^2^	*k*_2_ (g mg^−1^ min^−1^)	*q*_e_ (mg g^−1^)	*r*^2^
Red	0.0025	11.4	0.98	3.2 × 10^−4^	8.6	0.42
Yellow	0.003	7.9	0.96	9.2 × 10^−4^	5.7	0.63
Green	0.0021	9.8	0.98	8.1 × 10^−4^	5.9	0.45
Blue	0.0023	10	0.99	16 × 10^−4^	4.4	0.32

The pseudo first-order kinetic model is used when the rate of occupation of the adsorption sites is proportional to the number of unoccupied sites on the adsorbent.

## Conclusion

Our study focused on studying the biosorption of four different reactive dyes onto *L. pellucida* algae under various conditions, including time, pH, dye concentration, algae biomass, and temperature. Moreover, two isotherms, Langmuir and Freundlich, are applied. The biosorption kinetics are investigated according to pseudo-first and pseudo-second order. The biosorbent *L. pellucida* alga was characterized by FTIR and SEM tools. The results of FTIR spectra show that different functional groups were found on the *L. pellucida* surface, e.g., OH, C = O, and NH_*x*_. The SEM images before and after dye sorption show the blockage of the active site and pores of the surface by dye molecules. The biosorption results revealed that the adsorption system is affected by various conditions. The adsorption process is favorable in basic media (pH 8) and at room temperature (up to 30 °C). The results show a high removal percentage (more than 95%) at 120 min for all tested dyes. The biosorption of these four dyes followed the pseudo-1st-order paradigm. When the occupation rate of the adsorption sites is proportional to the quantity of vacant sites on the adsorbent, the pseudo-first-order kinetic model is employed.

## Data Availability

The datasets generated and analyzed during the current study are available from Mofida Makhlof and Hussein Khalaf.
